# Organ-wide profiling in mouse reveals high editing levels of Filamin B mRNA in the musculoskeletal system

**DOI:** 10.1080/15476286.2018.1480252

**Published:** 2018-07-31

**Authors:** Philipp Czermak, Fabian Amman, Michael F. Jantsch, Laura Cimatti

**Affiliations:** a Center of Anatomy and Cell Biology, Division of Cell Biology, Medical University of Vienna, Vienna, Austria; b Institute of Theoretical Biochemistry, University of Vienna, Vienna, Austria; c Max F. Perutz Laboratories, Department of Chromosome Biology, University of Vienna, Vienna, Austria

**Keywords:** Adenosine to inosine RNA editing, ADARs, Filamin B, mouse development, amplicon sequencing

## Abstract

Adenosine to inosine RNA editing in protein-coding messenger RNAs (mRNAs) potentially leads to changes in the amino acid composition of the encoded proteins. The mRNAs encoding the ubiquitously expressed actin-crosslinking proteins Filamin A and Filamin B undergo RNA editing leading to a highly conserved glutamine to arginine exchange at the identical position in either protein. Here, by targeted amplicon sequencing we analysed the RNA editing of Filamin B across several mouse tissues during post-natal development. We find highest filamin B editing levels in skeletal muscles, cartilage and bones, tissues where Filamin B function seems most important. Through the analysis of Filamin B editing in mice deficient in either ADAR1 or 2, we identified ADAR2 as the enzyme responsible for Filamin B RNA editing. We show that in neuronal tissues Filamin B editing drops in spliced transcripts indicating regulated maturation of edited transcripts. We show further that the variability of Filamin B editing across several organs correlates with its mRNA expression.

## 1. Introduction

RNA editing by adenosine deaminases acting on RNA (ADARs) converts adenosines (A) to inosines (I) in structured and double-stranded regions of RNAs [1]. With respect to their basepairing properties, inosines resemble guanosines. Consequently, A to I conversions can change the stability of double-stranded regions in RNA, alter splice sites, or lead to the recoding of mRNAs [2]. A to I editing is very abundant in mammals, e.g., in the human transcriptome there more than three million editing sites have been identified [3].

While the majority of editing events occurs in untranslated, repeat-containing regions of mRNAs, about 100 editing events are found in coding regions of mRNAs that lead to recoding of the mRNAs. Their evolutionary conservation further underscores the importance of these editing events [4]. In mammals, ADAR1 and ADAR2 are the two active editing enzymes identified today [5]. While ADAR1 primarily edits non-coding regions and is required to mark endogenous RNAs as ‘self’ [6], ADAR2 seems more involved in editing coding regions of mRNAs [7].

The majority of protein recoding editing events have been identified in the nervous system, where the editing-induced amino acid exchanges modulate the activity of the encoded proteins [8]. Outside the nervous system, one of the most prominent editing-induced recoding events occurs in filamins. Filamins are actin-binding proteins which link the actin network to cellular membranes and transmembrane receptors [9]. The interactions with a plethora of proteins link filamins to different cellular processes, such as signalling, transcriptional regulation, migration, and tissue organization [10]. In vertebrates, filamin A, B, and C (FLNA, FLNB, FLNC) are known. By homology, FLNC is most distant and exclusively expressed in a few tissues, such as heart, skeletal muscles and bladder [11,12]. In contrast, FLNA and FLNB are highly homologous and ubiquitously expressed [13]. FLNA and FLNB are built up of an N-terminal actin-binding domain which is followed by 24 immunoglobulin (Ig)-like repeats, interrupted by two hinge regions. Homo- and heterodimerization of FLNA and FLNB occur via their carboxyl-terminal Ig-like repeat [14]. A recent study showed that FLNA and FLNB expression is reciprocally regulated with opposing effects on actin dynamics and cell spreading [15].

In humans, mutations in filamin A and B give rise to developmental disorders. While Filamin A mutations show cardiac, neurological, skeletal and intestinal impairments, Filamin B mutations only affect joint and skeletal development (Online Mendelian Inheritance in Man, OMIM®. Johns Hopkins University, Baltimore, MD. MIM Number: *300017 and *603381, respectively). This difference is mirrored in the mouse, where the deletion of the *Flna* gene causes lethal heart and vascular defects [16,17] while the deletion of the *Flnb* gene leads to lethal skeletal malformations with bone fusions [18,19].

The pre-mRNAs encoding FLNA and FLNB undergo RNA editing leading to a glutamine (Q) to arginine (R) exchange at the identical position in either protein [7]. Editing of *Flna* RNA is conserved from birds to humans and is located in a highly interactive region of Filamins seemingly affecting multiple cellular functions. Our previous spatio-temporal analysis showed that *Flna* RNA editing in the mouse is highest outside of the nervous system, particularly in lungs, blood vessels and gastrointestinal tract [20].

In human and murine *Flnb* transcripts, a second editing event has been observed upstream of the known Q/R site [21,22]. In humans, this second editing site at chr3:58,156,064 (GRCh38/hg38) leads to a methionine (M) to valine (V) exchange. Interestingly, the corresponding position in the mouse is a genomically fixed guanosine. Instead, editing of adenosine chr14:7,936,041 (GRCm38/mm10) results in a serine (S) to a glycine (G) exchange in the murine *Flnb* protein (S/G site).

Here, we profile *Flnb* editing in mature mRNAs across several organs during post-natal development. Together, we show that editing of *Flnb* is highest in the musculoskeletal system where FLNB has been found to be most essential. Seemingly *Flnb* editing is independently regulated from editing of *Flna*. Furthermore, we confirm that ADAR2 is the enzyme responsible for *Flnb* RNA editing and that the *Flnc* transcript does not undergo A-to-I editing.

## 2. Results

To determine the distribution, abundance and developmental change of ADAR-mediated editing of filamin B mRNA we comprehensively quantified editing levels of this RNA in mouse organs at different time points during post-natal development using Illumina sequencing technology. The strategy applied to uniquely identify up to 96 multiplexed samples per sequencing round is outlined in Suppl. Figure 1. Briefly, a short PCR amplicon containing the *Flnb* Q/R editing site was obtained from cDNAs generated from different mouse tissues at birth (P0), 21 days (P21) and 120 days after birth (P120) in three biological replicates for each time point. The tissue origin and time point of every sample were marked by a combination of two 8 nucleotide-long barcodes, both on the 5ʹ- and 3ʹ-ends. A total of 200 samples was sequenced. Replicas of 19 tissue-derived samples at P0, 24 tissues at P21 and P120 together yielded 1.49 million reads. On average, a coverage of 7,450 reads was obtained per sample, varying from a minimum of 247 to a maximum of 7,990 reads per sample (median = 7,824 reads). Sequencing quality was assessed in every sample. Even at the lowest coverage the observed background sequencing error rate – in the range of one-tenth of a percent – did not affect the quantification of editing rates, which is higher by at least one order of magnitude (Suppl. Figure 2).

The A-to-I editing rate was determined as the number of reads with a guanosine (G) instead of the expected genomic adenosine (A) over the total number of reads in the sample. To verify the editing levels obtained by amplicon sequencing, a few samples were also tested by direct Sanger sequencing. As expected, amplicon sequencing was more sensitive to detect low editing rates while similar editing levels were detected at higher rates (Suppl. Figure 3).

**Figure 1.**
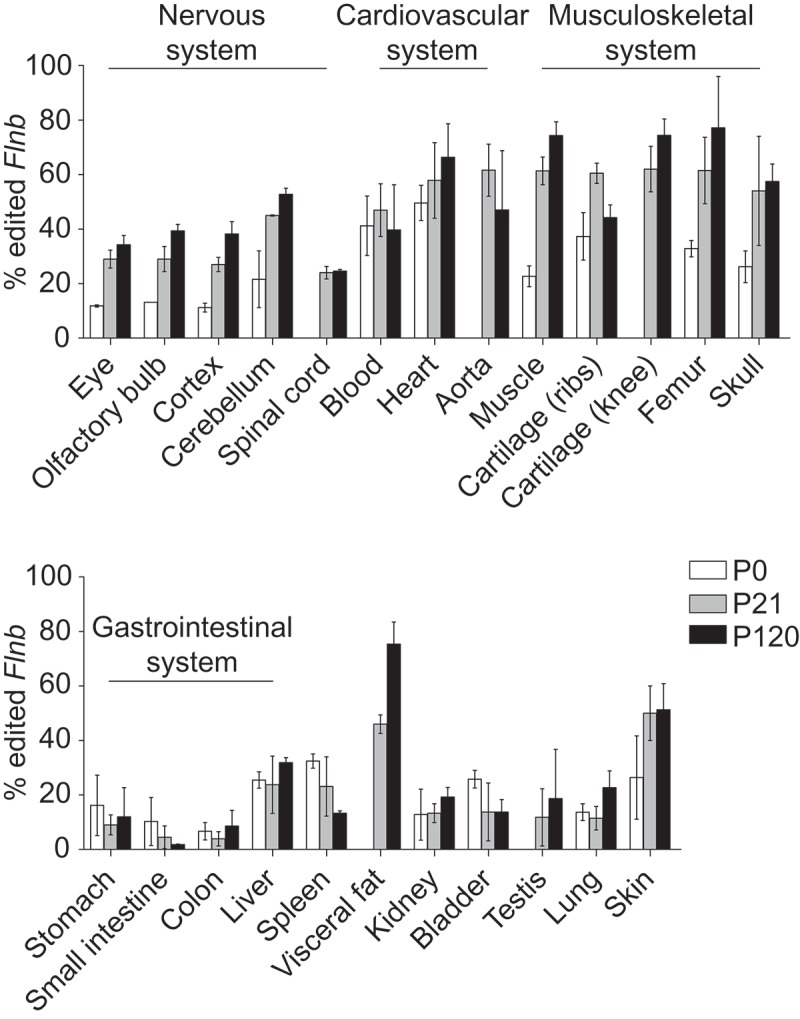
Flnb A-to-I editing levels across mouse tissues. Proportion of edited *Flnb* mRNA in mouse tissues at birth (P0), 21 and 120 days after birth (P21 and P120, respectively). *Flnb* editing increases with age and the highest editing levels are detected in the musculoskeletal apparatus (top panel). Data are plotted as mean ± standard deviation.

The A-to-I editing rate at the *Flnb* Q/R site (chr14:7,936,048) varied greatly amongst different mouse tissues, ranging from a minimum of 1.8% in small intestine in young mice (P21) to a maximum of 77.2% in femur in adult mice (P120) (Figure 1). As shown for other editing events, *Flnb* Q/R editing increased with age. Indeed, editing levels in tissues of young and adult mice were higher than at birth, with only few exceptions (spleen, gastrointestinal organs and bladder) where editing levels decreased or stayed constant with increasing age (Figure 1).

Within the nervous system highest *Flnb* editing levels were detected in cerebellum at any given time point increasing from 22% in newborns to 53% in P120 mice (Figure 1, top panel). While many editing substrates are most prominently edited in the brain [23] our analysis revealed elevated levels of *Flnb* RNA editing in non-brain tissues. Highest rates of Flnb editing were observed in the cardiovascular and musculoskeletal systems. The aorta of P21 mice showed 62% editing while the heart of P120 mice showed 66% editing of the *Flnb* Q/R site (Figure 1, top panel). Within the musculoskeletal system the skeletal striated muscle and cartilage of the hind limb showed more than 74% of A-G transitions in adult mice. In bones, femur (long bone) showed higher editing levels than skull (flat bone) with 77% and 57% A-G transitions at P120, respectively (Figure 1, top panel). Low levels of *Flnb* editing were detected along the gastrointestinal tract and in the excretory system. Interestingly, high levels of A-G transitions were reported for white visceral fat tissue (75.3%) and skin (51.3%) in adult mice, tissues previously appreciated to display no significant level of editing (Figure 1, bottom panel).

The recent publication from Tan et al. [24]. provides the most updated and comprehensive reference for global A-to-I RNA editing. The *Flnb* editing levels measured by us are highly similar to those reported in the corresponding mouse Genotype-Tissue Expression (GTEx) data set (Suppl. Figure 4(A)). However, the mouse editing levels differ greatly from those reported for human tissues, where *Flnb* RNA editing is in general lower than in the mouse (Suppl. Figure 4(B)).

**Figure 2.**
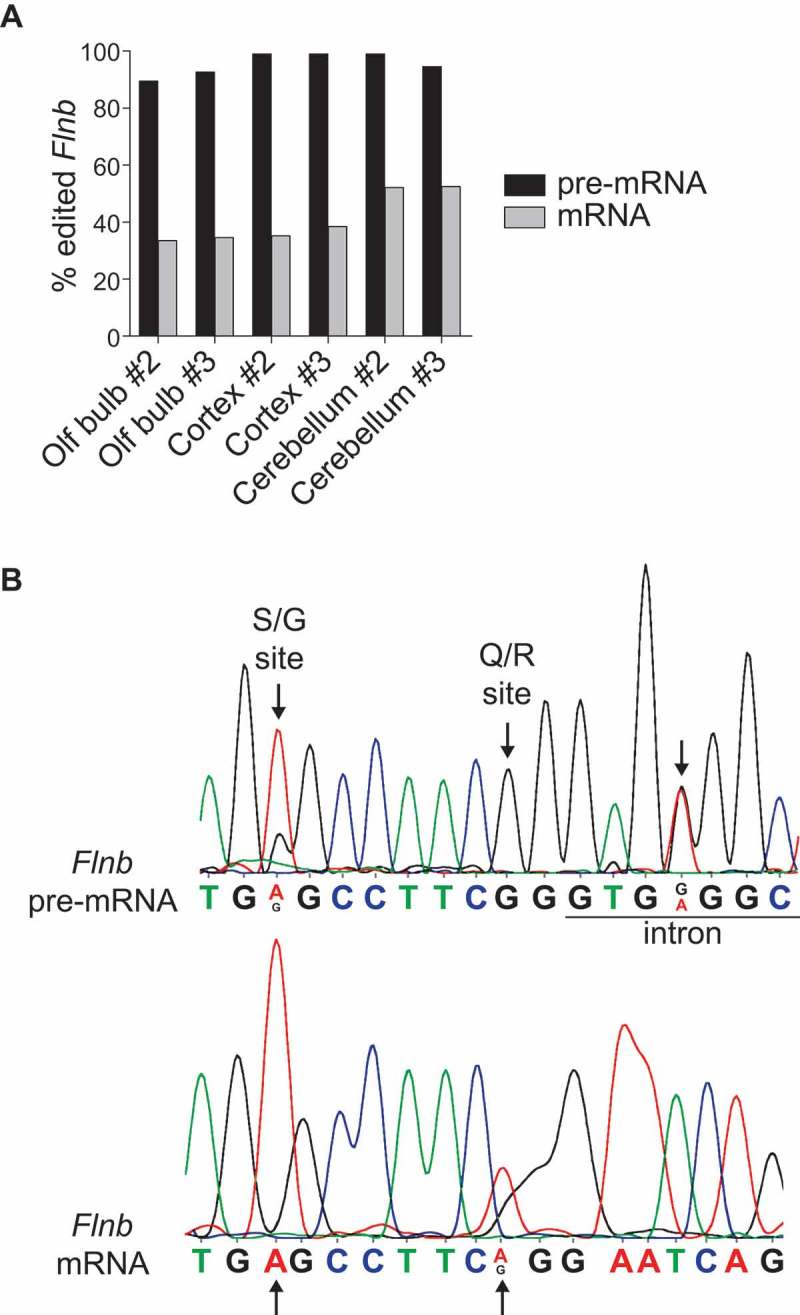
Flnb pre- and mRNA are differentially edited in mouse brain. A) Editing levels at the Q/R site of *Flnb* pre- and mRNA were measured by Sanger sequencing in two biological replicas of wild type olfactory bulb, cortex and cerebellum. In all samples unspliced Flnb pre-mRNA is highly edited, while only 30% to 50% of spliced *Flnb* mRNAs are edited. B) Chromatrograms showing *Flnb* pre-mRNA (top panel) and *Flnb* mRNA (bottom panel) in wild type cerebral cortex. Arrows point the S/G and Q/R editing sites in *Flnb* exon and the adenosine edited in the downstream intron.

The *Flnb* editing levels we observed in brain tissues differ from those of a previous study, where 95% *Flnb* editing was observed by RNA sequencing of whole-brain samples from several mouse strains [22]. In our study the applied amplicon sequencing targets exclusively spliced *Flnb* mRNA, whereas the RNA sequencing also captures unspliced and partially spliced pre-mRNA transcripts. To check whether the lower levels of *Flnb* editing detected by us could be due to the enrichment of mature transcripts, we compared A to G transitions by Sanger sequencing on both unspliced and spliced *Flnb* RNA transcripts amplified from the same tissue using specific primers. In all tested P120 brain samples editing at the Q/R site was above 80% in *Flnb* pre-mRNA transcripts, while editing frequency decreased to ~50% in spliced *Flnb* mRNA (Figure 2). RNA editing at the S/G site was also detectable in the *Flnb* pre-mRNA only (Figure 2(B)). These results suggest that edited Flnb transcripts are less efficiently spliced in mouse brain tissues. A closer look at the Flnb pre-mRNA chromatogram revealed an additional edited adenosine in the following intron (chr14:7,936,053) (Figure 2(B)) which is still part of the double stranded tract formed with the downstream editing complementary sequence (Suppl. Figure 5). Interestingly, this additional editable adenosine lies within the 5ʹ splice consensus sequence at position + 4 downstream of the exon-intron boundary, likely affecting splicing efficiency. To estimate the strength of the Flnb 5ʹ splice-site junction in edited and unedited transcripts we compared splice site sequence motifs by the maximum entropy (MaxENT) principle [25]. MaxENT scores for unedited transcripts, transcripts uniquely edited at the Q/R site or at the intronic adenosine and fully edited transcripts are shown in Table 1. Among the four possible transcripts unedited *Flnb* transcripts showed the strongest 5ʹ splice site while the presence of a single RNA editing event decreases splicing efficiency. By an additive effect, the presence of both edited nucleotides in the *Flnb* 5ʹ splice sites further reduces the likelihood of being spliced for fully edited *Flnb* transcripts.

**Table 1.** Maximum entropy scores for edited and unedited Flnb splice site. Using the maximum entropy model, the strength of the 5ʹ splice site at exon 41 in *Flnb* transcript was scored according to the editing status of its sequence. The fully unedited and edited sequences have the highest and lowest likelihood of being spliced, respectively.

Exonic sequence	Intronic sequence	MaxENT score
CAG	gtgagg	10.07
CGG	gtgagg	8.48
CAG	gtgggg	6.92
CGG	gtgggg	3.69

RNA sequencing data recently obtained from the cerebral cortex of ADAR1 or ADAR2 knockout (KO) mice allowed us to determine which of the two functional RNA editing enzymes is responsible for *Flnb* editing. While in ADAR1 KO cortex *Flnb* editing was detected in about 30% of the reads, no A-G transitions were detected on *Flnb* RNA in the cortex of ADAR2 KO mice (Table 2). To confirm that ADAR2 is the enzyme responsible for *Flnb* editing, more tissues were isolated from wild type and ADAR2 knockout mice and *Flnb* editing levels analysed by Sanger sequencing. While Flnb editing was clearly detectable in all wild type tissues, no edited *Flnb* transcript was detected in the ADAR2 knockout samples (Table 3).

**Table 2.** Flnb A-to-I editing in the cerebral cortex of ADAR1 and ADAR2 knockout mice. Quantification of A-to-I editing events at the Flnb Q/R site by RNAseq data obtained from ADAR1 and ADAR2 knockout mice. Adenosine to guanosine transitions were detected in ADAR1^-/-^ but not in ADAR2^-/-^ cortices (n = 3).

	ADAR1	ADAR2
Wild type	39.3%	34.7%
Knockout	31.3%	0%

**Table 3.** Flnb A-to-I editing in ADAR2 knockout tissues. To confirm that ADAR2 enzyme is responsible for *Flnb* RNA editing different tissues were isolated from wild type and ADAR2 knockout mice and editing checked by Sanger sequencing. Adenosine to guanosine transitions were detected in ADAR2 wild type tissues only.

ADAR2	wild type	knockout
Cortex	30,9%	0%
Cerebellum	42,7%	0%
Heart	62,3%	0%
Skeletal muscle	68,5%	0%
Femur	50,9%	0%
Adipose tissue	62,1%	0%

**Figure 3.**
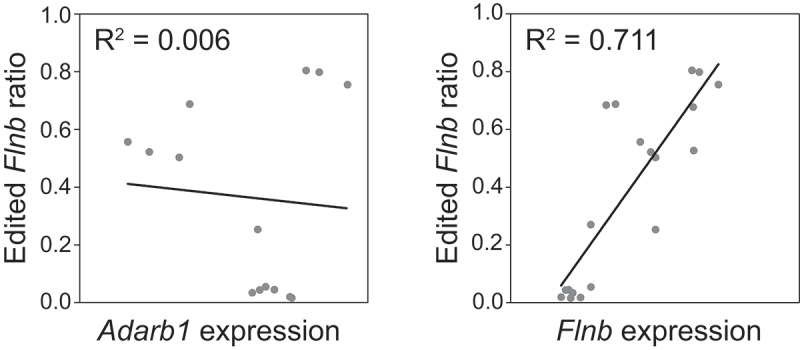
Flnb RNA editing does not correlate with ADAR2 but with Flnb expression. *Flnb* editing (y axis) was measured by amplicon sequencing and *Adarb1* (left panel) or *Flnb* (right panel) mRNA expression was quantified by qPCR on the same samples. The variation in *Flnb* editing among samples does not correlate with *Adarb1* expression (n = 14). Instead, about 70% of the observed variation in *Flnb* editing could be positively correlated to Flnb mRNA expression (n = 19).

To test whether *Flnb* Q/R editing level and ADAR2 expression correlate, quantitative PCR was performed on representative samples from adult wild type mice. As shown in Figure 3, there is no linear correlation between the proportion of *Flnb* edited transcripts and ADAR2 mRNA expression in the same tissue. Next, *Flnb* mRNA expression was measured by quantitative PCR in samples from adult mice. A positive correlation is apparent (R^2^ = 0.7113) between the expression level of *Flnb* mRNA and its editing frequency (Figure 3).

**Figure 4.**
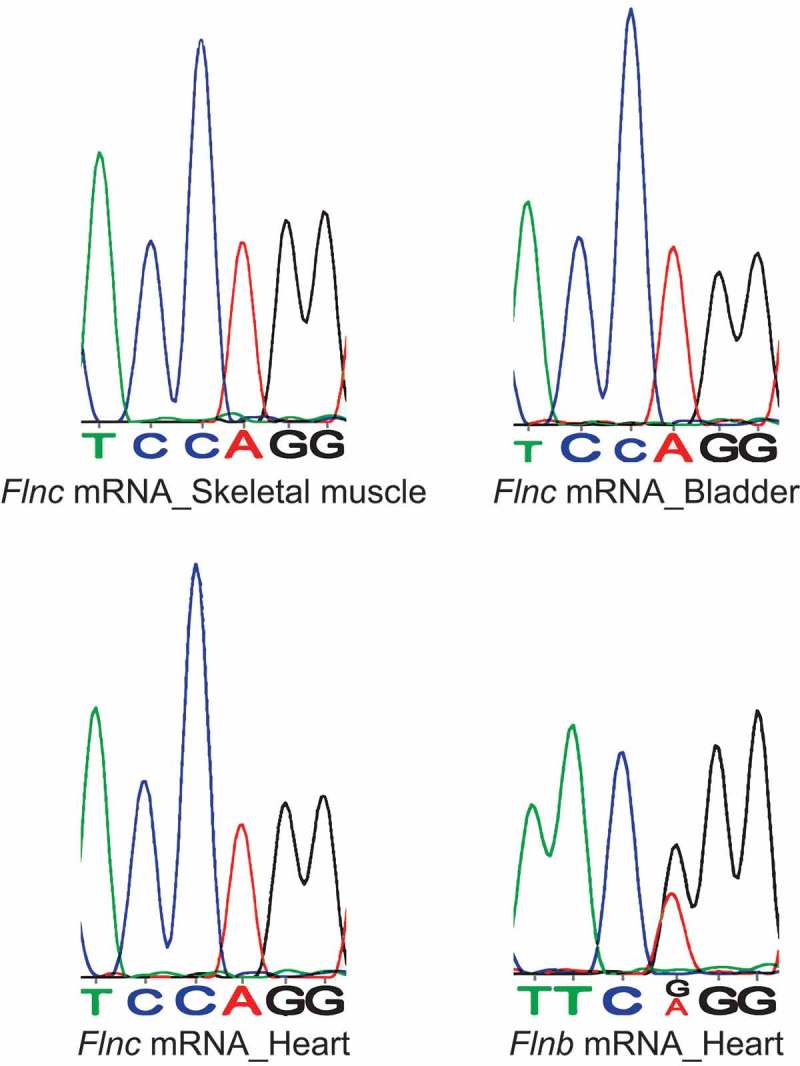
Flnc mRNA does not undergo A-to-I editing. Chromatrograms showing no evident A-G transition in *Flnc* mRNA amplified from wild type heart, skeletal muscle and bladder. The chromatrogram for Flnb mRNA in wild type heart is shown for comparison.

Despite a low degree of sequence homology, murine Filamin C (Flnc) mRNA and amino acid sequences overlap the Q/R editing site on murine Flna and Flnb. To check if *Flnc* mRNA also undergoes A-to-I editing at this overlapping position (chr6:29,457,557) amplicons from wild type adult mouse tissues where *Flnc* is mainly expressed [11,12] were analysed by Sanger sequencing. No A-G transitions were detected in heart, skeletal muscle or bladder samples (Figure 4).

## 3. Discussion

Previous studies have shown a gradual increase of A-to-I editing levels for protein-coding transcripts during mammalian neuronal development [26,27] in the presence of constant protein levels of ADAR1 and ADAR2 [28]. Recently, it was shown that in developing mouse neurons increased nuclear localization of ADAR2 might contribute to this phenomenon [29] together with protein modifications on ADARs and regulatory factors [30,31]. We previously showed that a gradual increase in editing is also found for filamin A, in both neuronal and non-neuronal tissues [20]. Here, we confirmed that also levels of *Flnb* editing increase with age in both nervous and non-nervous tissues.

The highest *Flnb* editing rates were detected outside the nervous system where most other protein-coding ADAR targets had been identified [23]. However, abundant editing outside the nervous system had also been found for *Flna* [20]. Interestingly, major differences between *Flna* and *Flnb* RNA editing profiles can be found. For instance, *Flna* RNA is highly edited in stomach and colon while *Flnb* editing is very low in all segments of the digestive tract. This suggests a likely functional role for *Flna* editing in the gastrointestinal apparatus.

Instead, *Flnb* editing is high in both the vascular and musculoskeletal systems. This finding is interesting as *Flnb* knockout mice show impaired development of the microvasculature and skeletal system with bone and joint fusions [18,19]. This also suggests a link between A-to-I editing of the *Flnb* transcript and the main function of the encoded protein. It should also be noted that all components of the musculoskeletal system show high *Flnb* editing levels.

The developmental origin of bones is heterogeneous. Craniofacial bones develop from the embryonic ectoderm cell layer while the limb skeleton generates from the mesoderm layer. Still both types of bones showed comparable high Flnb editing levels.

Surprisingly, we observed a high *Flnb* editing frequency in white abdominal fat, especially in adult mice. To our knowledge this is the first time *Flnb* editing is measured in adipose tissue. Further investigation would be needed to clarify the role of *Flnb* editing in fat where it might be linked to both metabolism and hormonal production.

Within the central nervous system the highest *Flnb* editing levels are detected in the cerebellum in agreement with recently published data [24]. Interestingly, the overall editing frequency observed in all neuronal tissues is inconsistent with a previous study conducted on whole-brain samples from several laboratory mouse strains [22]. Beyond the difference in brain sampling, in this case editing levels were determined by RNA sequencing, thus in both *Flnb* pre-mRNA and mRNA. In the present study editing levels were determined exclusively on spliced *Flnb* mRNA. Indeed, we could verify that Flnb editing was elevated in pre-mRNA compared to mRNA in all samples tested.

Moreover, apart from the already known S/G and Q/R sites we observed an editing site located in the intronic 5ʹ splice site of the *Flnb* pre-mRNA. Therefore, we speculated that the presence of two editable nucleotides in the splice site consensus sequence could affect splicing efficiency. Comparing the intron-exon junction of unedited, partially edited or fully edited *Flnb* transcripts to the consensus 5′ splice site sequences clearly showed that RNA editing decreases the probability of a *Flnb* transcript of being spliced. It seems therefore plausible that edited *Flnb* transcripts are less efficiently spliced, a phenomenon already reported by us and others [32,33].

Terajima et al. recently reported that in the mouse liver ADAR2 is the enzyme responsible for editing of the *Flnb* Q/R site [34]. This contrasts with another studies reporting that *Flnb* editing is catalysed by both ADAR enzymes [35]. In this study using amplicon sequencing we could confirm that also in the mouse cerebral cortex the enzyme responsible for editing of the *Flnb* transcript is ADAR2. In fact, no edited adenosine and no compensatory activity from ADAR1 enzyme were detected at the *Flnb* Q/R site in ADAR2 knockout samples. Next, by Sanger sequencing we confirmed that ADAR2 is the enzyme responsible for *Flnb* RNA editing in other tissues of both neuronal and non-neuronal origin. Nevertheless, we observed no direct correlation between ADAR2 mRNA expression and *Flnb* editing at the Q/R site, suggesting that *Flnb* editing does not depend on the stoichiometry of the enzyme-substrate ratio. Instead, *Flnb* mRNA expression seemingly correlates with the variability of *Flnb* editing levels across mouse tissues. This finding is in agreement with previous studies where similar variabilities in editing levels were observed [36]. At this point we cannot exclude that ADAR2 activity on this specific transcript could be at least in part a regulated process. As there is no correlation between *Flnb* editing and ADAR2 expression, editing of *Flnb* could possibly be affected by competing RNA-binding proteins or editing regulators [24,30,31,37]. This idea is also supported by the lack of correlation of editing levels in *Flna* and *Flnb* RNAs which are both edited by ADAR2. Our data also show that editing levels in pre-mRNA and mRNA differ. Thus, levels of editing in mature mRNAs may also be regulated by differential splicing. Moreover, *Flnb* editing has been shown to be altered in esophageal and liver carcinomas due to the increased activity of ADAR1 indicating that this enzyme may also target *Flnb*, at least under specific conditions [38,39]. Clearly, further investigations concerning the modulation of *Flnb* RNA editing will be required.

Eventually, despite a high degree of transcript sequence homology in the region corresponding to the *Flna* and *Flnb* editing sites, no adenosine to guanosine transition was detected in *Flnc* mRNA.

In summary, with this study we confirmed that in mouse not only filamin A but also filamin B mRNA is highly edited outside the nervous system. The tissue specificity of the editing profiling of these two filamins highlights both common and different traits, suggesting a distinctive role for the RNA editing of these two cytoskeletal proteins. In particular, Flnb editing is elevated in the very same musculoskeletal tissues which are mostly affected in Flnb-associated human diseases and in Flnb-deficient mice. Indeed, uncovering the functional role of Flnb editing will be the next experimental challenge.

## 4. Materials and methods

### 4.1. Mice

For *Flnb* RNA editing profiling male wild type (C57Bl/6J) mice were sacrificed and dissected organs were snap frozen in liquid nitrogen.

Cerebral cortices of rescued ADAR1 knockout [6] and ADAR2 knockout [32] mice were dissected at day 15 after birth and snap frozen in liquid nitrogen; wild type littermates were used as control. For further analysis on different tissues adult (> P120) ADAR2 knockout mice were used.

### 4.2. RNA isolation

Tissues were homogenized in peqGOLD TriFast™ reagent (PEQLAB Biotechnologie GmbH, Germany) and total RNA was extracted following the manufacturer’s instructions. For *Flnb* amplicon sequencing, triplicates of tissues from three C57BL/6J mice per time point (P0, P21 and P120) were analyzed.

### 4.3. cDNA synthesis

Aliquots of total RNA were digested with DNase I (Thermo Fisher Scientific) in the presence of Ribolock RNase inhibitor (Thermo Fisher Scientific) following the manufacturer’s instructions. cDNAs were synthesized using the Revert Aid reverse transcriptase (Thermo Fisher Scientific) in the presence of random hexamers. To detect possible genomic DNA contamination a control reaction lacking the reverse transcriptase was performed in parallel for every sample.

### 4.4. Amplicon generation and illumina sequencing

Filamin B fragments were amplified in a 25 cycle Polymerase Chain Reaction (PCR) using the Q5® High-Fidelity DNA Polymerase (New England Biolabs Inc.). Primers were designed on different Filamin B mRNA exons, close to the editing site (underlined sequence) and contained part of the Illumina Adaptor Sequence (in italics).

Forward primer: 5ʹ-*ACACGACGCTCTTCCGATCT*TCGCTGCCTCACTGTTCTGAG-3ʹ

Reverse primer: 5ʹ-*CAGACGTGTGCTCTTCCGATCT*CGCTGGCTGGTTAACTTTTAATC-3ʹ

PCR products (100 nucleotide long) were purified on silica columns (Wizard® SV Gel and PCR Clean-Up System kit, Promega). The eluted samples were used for a second round of PCR (15 cycles) with the NEB Next® Multiplex Oligos for Illumina® – Dual Index Primers Set 1 (New England Biolabs Inc.). PCR products were run on a 2% agarose gel and bands at the expected height (194 nucleotides) were cut and purified on silica columns. The DNA concentration of the eluted samples was quantified by fluorimetric assay (Invitrogen™ Qubit™ 3.0 Fluorometer, Thermo Fisher Scientific) and samples were pooled accordingly.

The overall process of cluster generation, sequencing, image processing, demultiplexing, and quality score calculation was performed at the VBCF NGS Unit (www.vbcf.ac.at).

Obtained reads were mapped against the full-length amplicon sequence using bwa mem [40] with default settings and base occurrence was deduced using bam-readcount (https://github.com/genome/bam-readcount) with the minimal base quality parameter and the minimal read mapping quality set to 20. All mouse genome coordinates mentioned refer to the genome assembly GRCm38/mm10.

### 4.5. Quantitative PCR

The expression of ADAR2 mRNA or Flnb pre- and mRNA was quantified by real time PCR (qPCR) on a selection of P120 tissues. qPCR was performed using the GoTaq qPCR master mix (Promega), the CFX Connect™ Real-Time PCR Detection System (Bio-Rad Laboratories, Inc) and the following primers:

Flnb mRNA forward: 5ʹ-CAGCCCTTACCTGGTGCCCGT-3ʹ

Flnb mRNA reverse: 5ʹ-CTTTGCATCAATCTTCCCTTTCG-3ʹ

Flnb pre-mRNA forward: 5ʹ-GTAACTACGAGGTGTCTATCAAGT-3ʹ

Flnb pre-mRNA reverse: 5ʹ-GATGGATGCTTCCTTGTGCC-3ʹ

ADAR2 forward: 5ʹ-CTTGCCCTGAAGGAGTTTTG-3ʹ

ADAR2 reverse: 5ʹ-TCAGTGCTGCTGGAACTCAT-3ʹ

GAPDH forward: 5ʹ- AATGTGTCCGTCGTGGATCT-3ʹ

GAPDH reverse: 5ʹ- CCCTGTTGCTGTAGCCGTAT-3ʹ

Expression levels were calculated by the ΔCt method using GAPDH as a reference gene.

A-to-I editing of Flnc mRNA was analyzed by Sanger sequencing of PCR amplicons obtained with the following primers:

Flnc forward: 5ʹ- CTCCCTCTCAGACGACGCTC-3ʹ

Flnc reverse: 5ʹ- GCATCAATCACACCACGTGC-3ʹ

### 4.6. Maximum entropy scores

Splicing scores were obtained using the MaxEntScan::score5ss software available here:

http://genes.mit.edu/burgelab/maxent/Xmaxentscan_scoreseq.html

According to the model based on the maximum entropy (MaxENT) principle [25] a log-odd ratio (MaxENT score) is assigned to a 9-mer sequence (3 exonic bases followed by 6 intronic bases). The higher the score, the higher the probability that the sequence is a true splice site or, given two sequences of differing scores, the higher scoring sequence has a higher likelihood of being used for splicing.
