# Supplementary material for: Organ-wide profiling in mouse reveals high editing levels of Filamin B mRNA in the musculoskeletal system
Source: RNA Biol. 2018 Jul 31;15(7):877–85. doi: 10.1080/15476286.2018.1480252 (PMC6161736; doi:10.1080/15476286.2018.1480252)
Supplement: Supplemental Material [file krnb-15-07-1480252-s001.zip › Supplementary information/Czermak et al_Suppl Figures.pptx]

## Slide 1
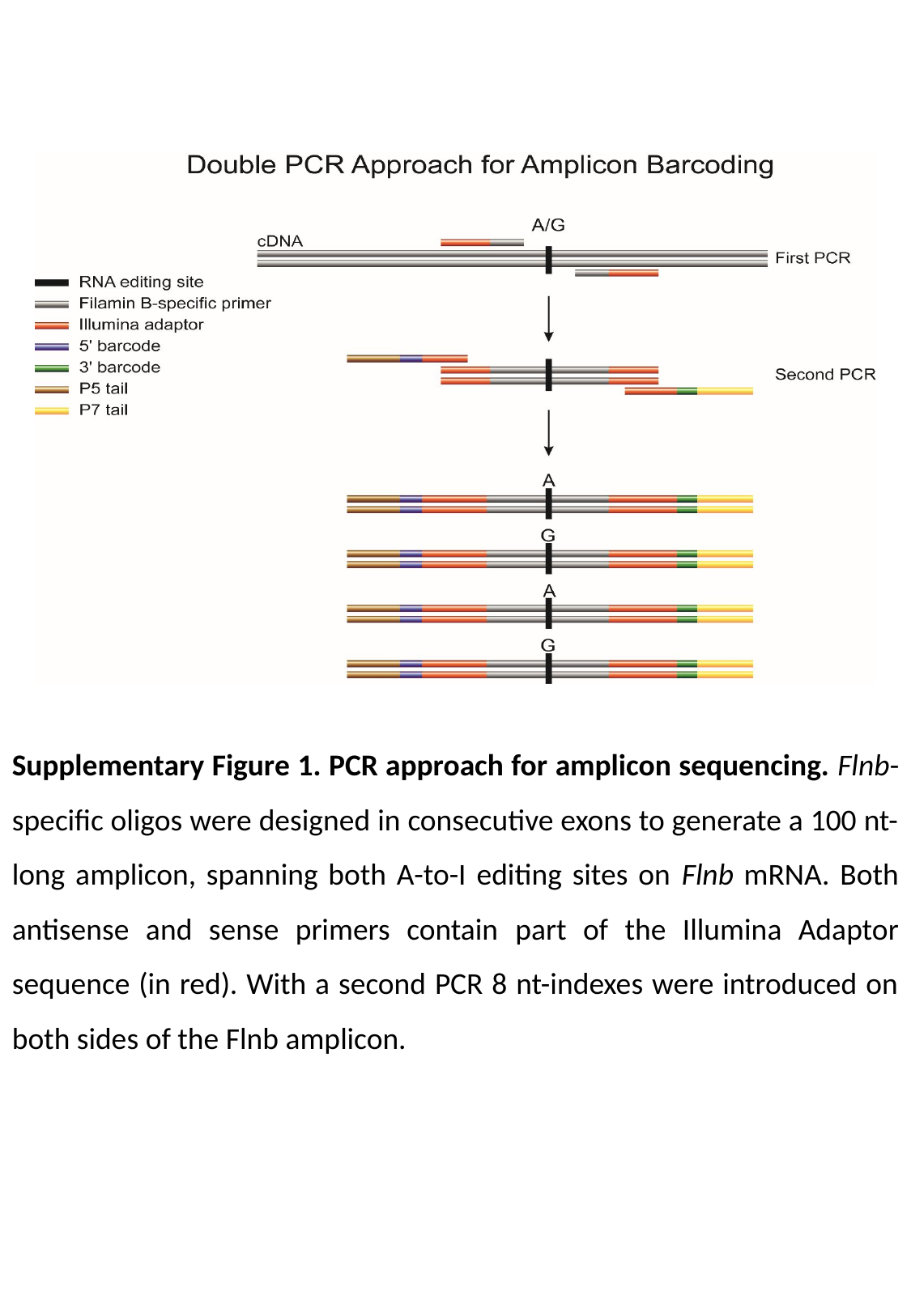

Supplementary Figure 1. PCR approach for amplicon sequencing. Flnb-specific oligos were designed in consecutive exons to generate a 100 nt-long amplicon, spanning both A-to-I editing sites on Flnb mRNA. Both antisense and sense primers contain part of the Illumina Adaptor sequence (in red). With a second PCR 8 nt-indexes were introduced on both sides of the Flnb amplicon.

## Slide 2
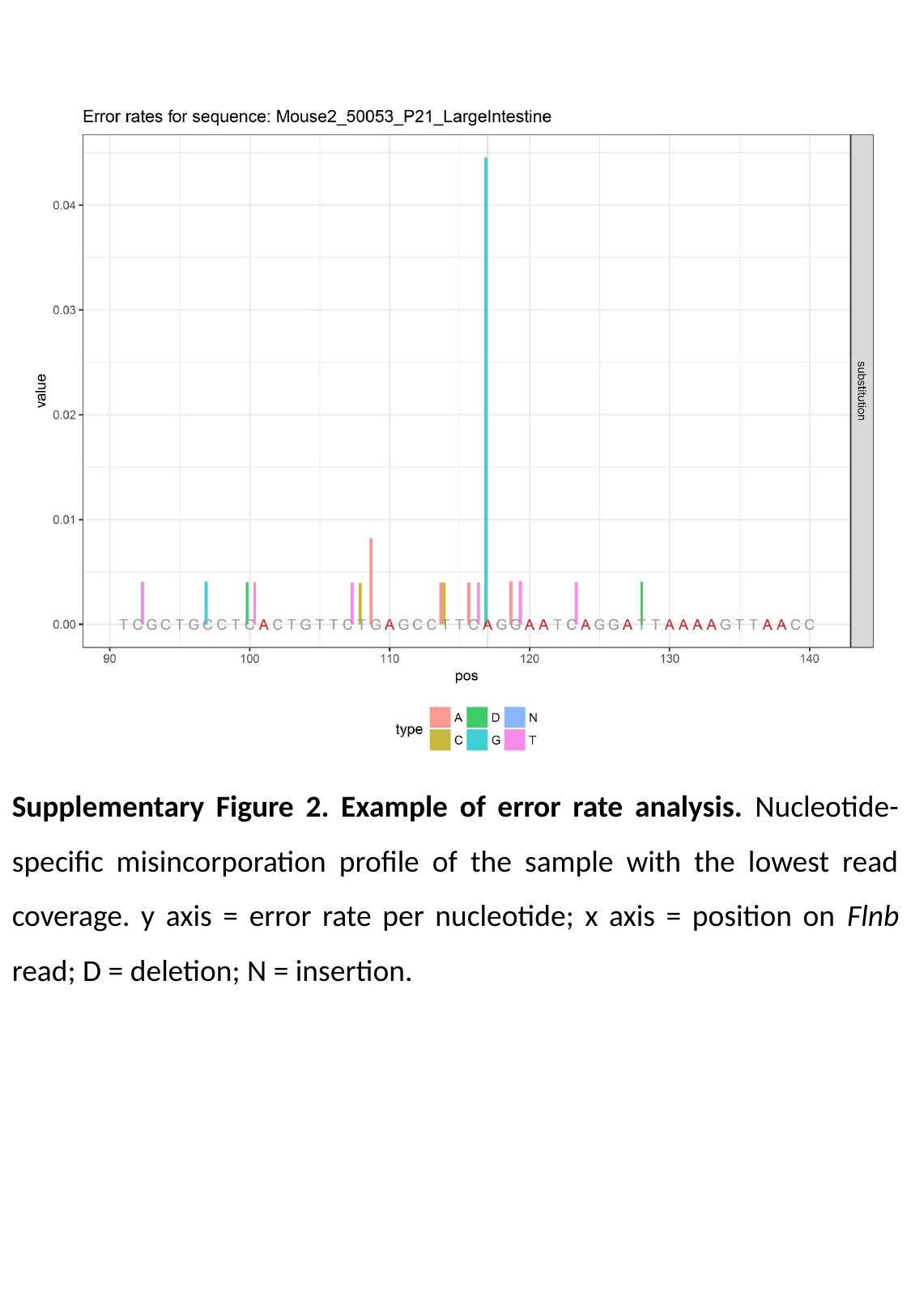

Supplementary Figure 2. Example of error rate analysis. Nucleotide-specific misincorporation profile of the sample with the lowest read coverage. y axis = error rate per nucleotide; x axis = position on Flnb read; D = deletion; N = insertion.

## Slide 3
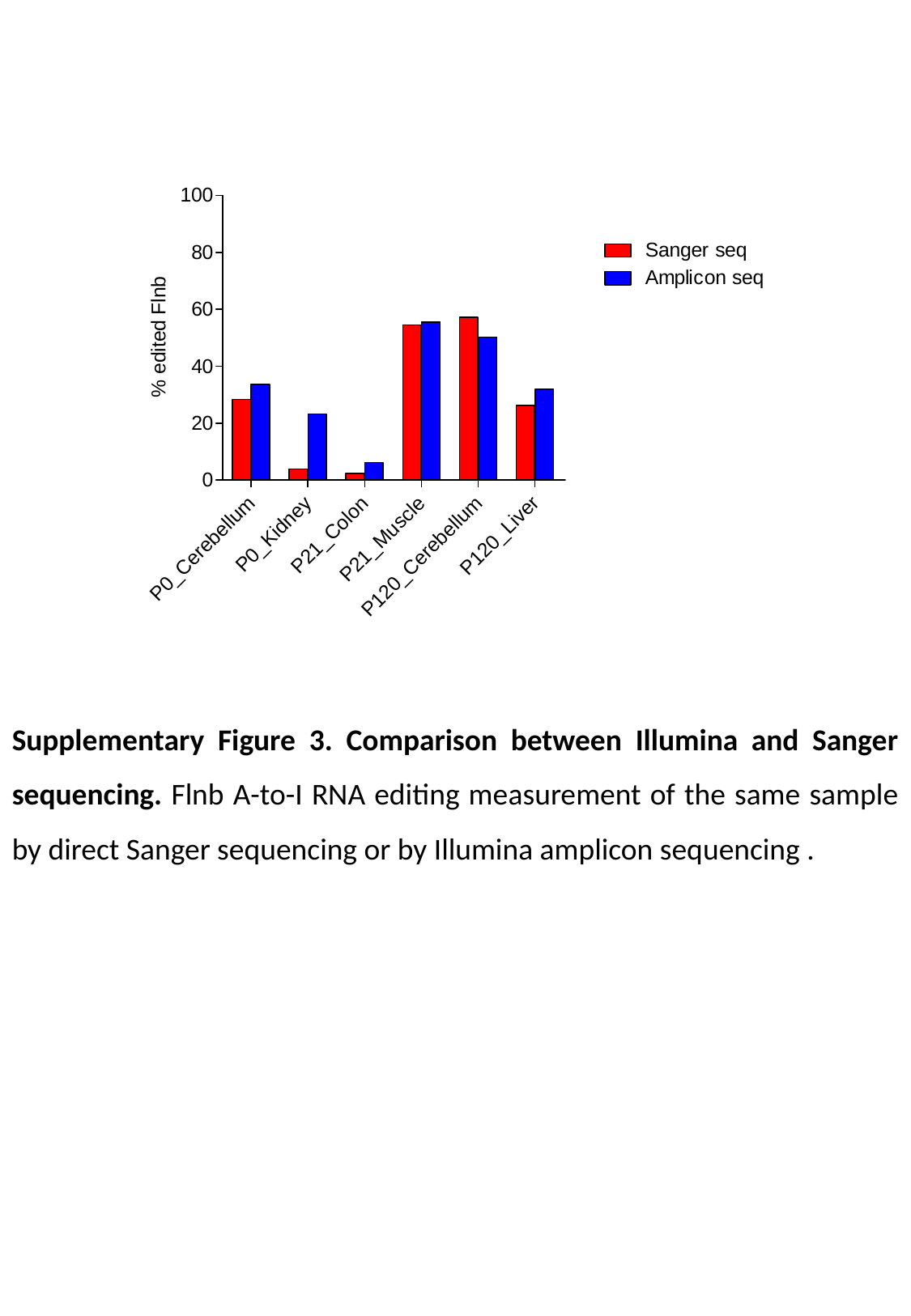

Supplementary Figure 3. Comparison between Illumina and Sanger sequencing. Flnb A-to-I RNA editing measurement of the same sample by direct Sanger sequencing or by Illumina amplicon sequencing .

## Slide 4
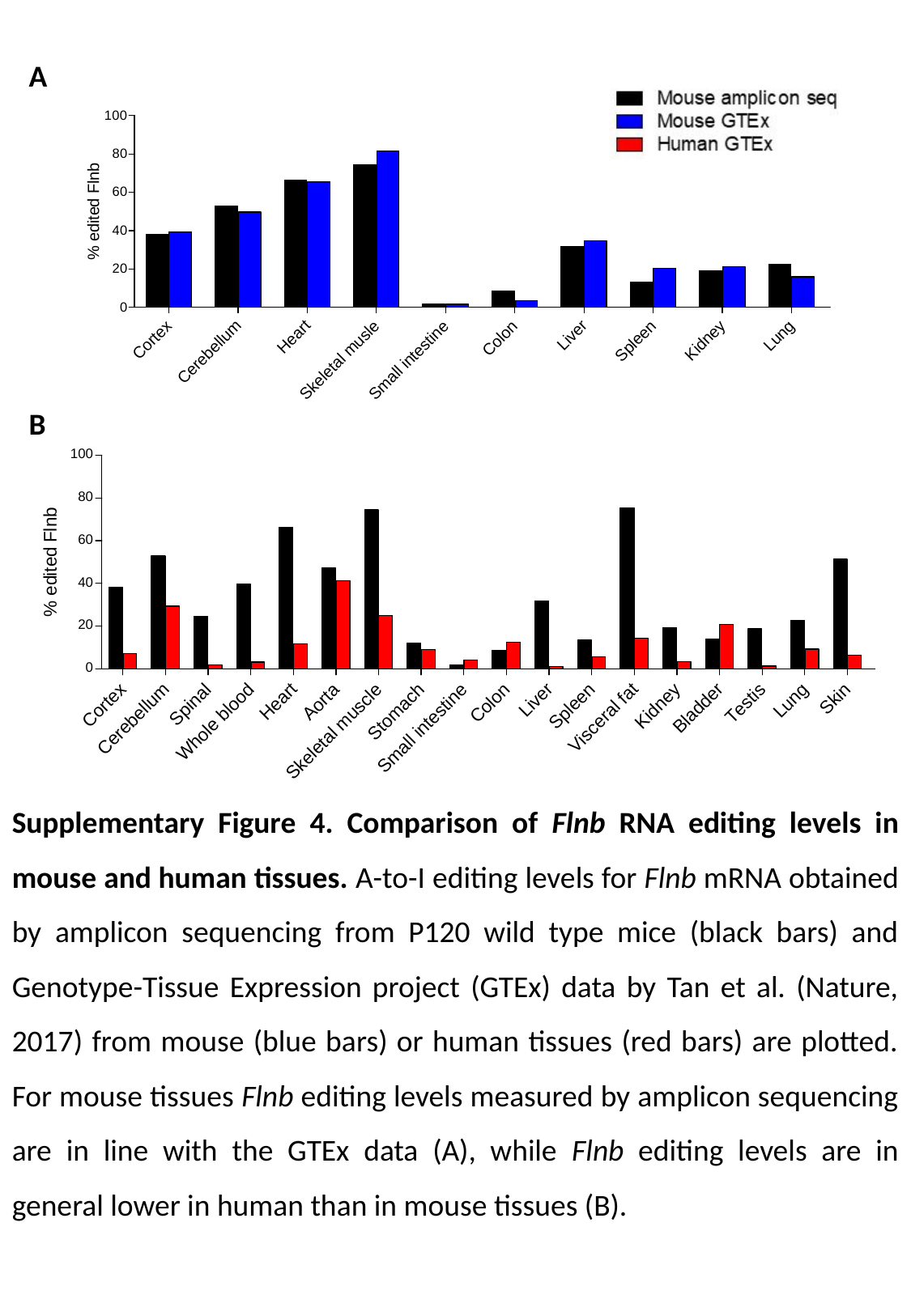

A
B
Supplementary Figure 4. Comparison of Flnb RNA editing levels in mouse and human tissues. A-to-I editing levels for Flnb mRNA obtained by amplicon sequencing from P120 wild type mice (black bars) and Genotype-Tissue Expression project (GTEx) data by Tan et al. (Nature, 2017) from mouse (blue bars) or human tissues (red bars) are plotted. For mouse tissues Flnb editing levels measured by amplicon sequencing are in line with the GTEx data (A), while Flnb editing levels are in general lower in human than in mouse tissues (B).

## Slide 5
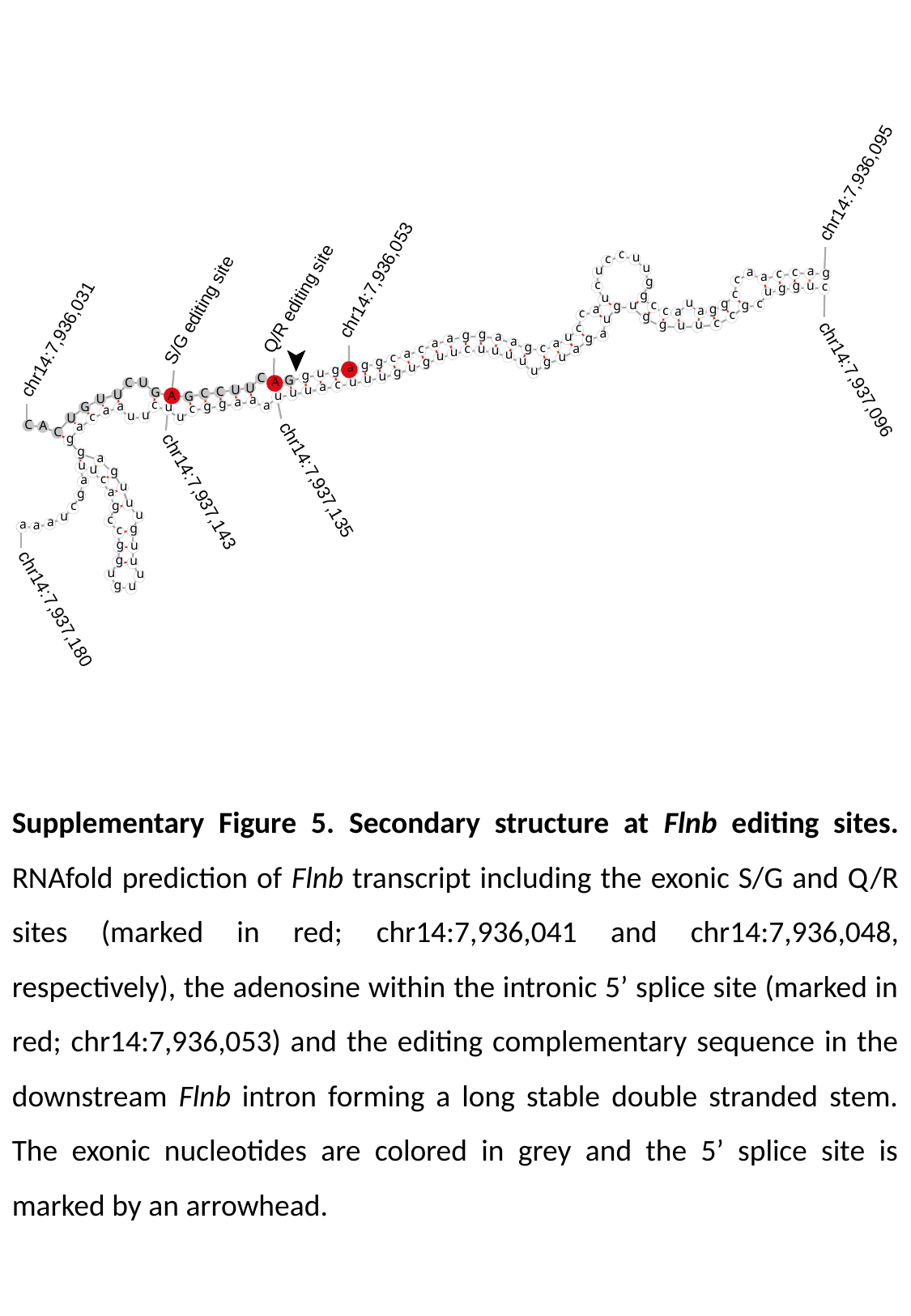

Supplementary Figure 5. Secondary structure at Flnb editing sites. RNAfold prediction of Flnb transcript including the exonic S/G and Q/R sites (marked in red; chr14:7,936,041 and chr14:7,936,048, respectively), the adenosine within the intronic 5’ splice site (marked in red; chr14:7,936,053) and the editing complementary sequence in the downstream Flnb intron forming a long stable double stranded stem. The exonic nucleotides are colored in grey and the 5’ splice site is marked by an arrowhead.
