# Supplementary material for: Organ-wide profiling in mouse reveals high editing levels of Filamin B mRNA in the musculoskeletal system
Source: RNA Biol. 2018 Jul 31;15(7):877–85. doi: 10.1080/15476286.2018.1480252 (PMC6161736; doi:10.1080/15476286.2018.1480252)
Supplement: Supplemental Material [file krnb-15-07-1480252-s001.zip › Supplementary information/Czermak et al_Tables.docx]

**Table 1. Maximum entropy scores for edited and unedited *Flnb* splice site.** Using the maximum entropy model, the strength of the 5’ splice site at exon 41 in *Flnb* transcript was scored according to the editing status of its sequence. The fully unedited and edited sequences have the highest and lowest likelihood of being spliced, respectively.

| **Exonic sequence** | **Intronic sequence** | **MaxENT score** |
| --- | --- | --- |
| CAG | gtgagg | 10.07 |
| CGG | gtgagg | 8.48 |
| CAG | gtgggg | 6.92 |
| CGG | gtgggg | 3.69 |

**Table 2. *Flnb* A-to-I editing in the cerebral cortex of ADAR1 and ADAR2 knockout mice.** Quantification of A-to-I editing events at the Flnb Q/R site by RNAseq data obtained from ADAR1 and ADAR2 knockout (KO) mice. Adenosine to guanosine transitions were detected in ADAR1^-/-^ but not in ADAR2^-/-^ cortices (n = 3).

|  | **ADAR1** | **ADAR2** |
| --- | --- | --- |
| **Wild type** | 39.3 % | 34.7 % |
| **Knockout** | 31.3 % | 0 % |

**Table 3. *Flnb* A-to-I editing in ADAR2 knockout tissues.** To confirm that ADAR2 enzyme is responsible for *Flnb* RNA editing different tissues were isolated from wild type and ADAR2 knockout mice and editing checked by Sanger sequencing. Adenosine to guanosine transitions were detected in ADAR2 wild type tissues only.

| **ADAR2** | **wild type** | **knockout** |
| --- | --- | --- |
| Cortex | 30,9 % | 0 % |
| Cerebellum | 42,7 % | 0 % |
| Heart | 62,3 % | 0 % |
| Skeletal muscle | 68,5 % | 0 % |
| Femur | 50,9 % | 0 % |
| Adipose tissue | 62,1 % | 0 % |
